# Testing multi-scale processing in the auditory system

**DOI:** 10.1038/srep34390

**Published:** 2016-10-07

**Authors:** Xiangbin Teng, Xing Tian, David Poeppel

**Affiliations:** 1 Department of Psychology, New York University, New York, NY, USA; 2 New York University Shanghai, Shanghai, 200122 China; 3 NYU-ECNU Institute of Brain and Cognitive Science at NYU Shanghai, Shanghai, 200122 China; 4 Department of Neuroscience, Max-Planck Institute, Frankfurt, Germany

## Abstract

Natural sounds contain information on multiple timescales, so the auditory system must analyze and integrate acoustic information on those different scales to extract behaviorally relevant information. However, this multi-scale process in the auditory system is not widely investigated in the literature, and existing models of temporal integration are mainly built upon detection or recognition tasks on a single timescale. Here we use a paradigm requiring processing on relatively ‘local’ and ‘global’ scales and provide evidence suggesting that the auditory system extracts fine-detail acoustic information using short temporal windows and uses long temporal windows to abstract global acoustic patterns. Behavioral task performance that requires processing fine-detail information does not improve with longer stimulus length, contrary to predictions of previous temporal integration models such as the multiple-looks and the spectro-temporal excitation pattern model. Moreover, the perceptual construction of putatively ‘unitary’ auditory events requires more than hundreds of milliseconds. These findings support the hypothesis of a dual-scale processing likely implemented in the auditory cortex.

Natural sounds, music, and vocal sounds have a rich temporal structure over multiple timescales^1^,^2^,^3^,^4^,^5^,^6^, and behaviorally relevant acoustic information is usually carried on more than one timescale. For example, speech conveys linguistic information at several scales: 20–80 ms for phonemic information, 100–300 ms for syllabic information, and more than 1000 ms for intonation information^1^. Bird song, similarly, also includes information on one timescale around 10–30 ms and another one around 500–700 ms^5^. Therefore, auditory processing requires sounds to be integrated temporally at different scales to extract critical perceptual information.

The temporal integration of sound is a fundamental property of hearing. Integrating information over time, as we define it operationally here, serves as a basic information accumulation process in the auditory system to guarantee that enough information is supplied for extracting meaningful regularities on a certain timescale. It has been demonstrated in various experiments, using different types of signals, that perceptual thresholds typically decrease as sound duration increases^7^,^8^. Such a trading relationship between sound duration and perceptual performance suggests that the auditory system accumulates information over time to get better estimates of auditory signal characteristics. Visual perception, especially in dynamic contexts, also incorporates such temporal integration processes^9^. As natural sounds contain information on multiple timescales, fine-grained (informally speaking, temporally ‘local’) information usually exists on a shorter timescale and requires a fast integration process, while more ‘global’ information occupies a larger timescale and a longer integration window is consequently needed. Therefore, by hypothesis, a single integration process may not suffice for the variety of auditory tasks that the system successfully solves.

The existing literature argues that the auditory system works over several distinct time ranges - instead of in a unitary manner across the (intuitively simpler and conceptually appealing) continuum of temporal variation^8^,^10^,^11^,^12^,^13^,^14^. Previous models of temporal integration typically assume leaky integration with a time constant of hundreds of milliseconds, based on studies such as loudness summation^15^,^16^, signal detection in noise^17^,^18^, and temporal integration at threshold^13^,^19^,^20^,^21^,^22^, in which listeners’ performance increases continuously with sound duration and typically achieves a plateau above 200 ms. These models, however, are not compatible with the range of results that demonstrate the high temporal resolution of the auditory system, such as in modulation detection^23^, gap detection^24^, and non-simultaneous masking^25^. In those studies, perceptual thresholds from 2–30 ms are the norm. This incompatibility has led to the formulation of the resolution-integration paradox^8^,^26^.

To resolve this paradox, Viemeister (1991) proposed a ‘multiple-looks’ model: the continuous sound is sampled discretely at a high rate (e.g. with integration windows of ~5 ms), and the samples are stored and concatenated/integrated for further processing. The multiple-looks model suggests that temporal integration is a process of multiple timescales instead of one, but the longer timescale is assembled on the basis of concatenated short samples. Moore (2003) further relates this concept to the internal representation of sounds, calculated as spectro-temporal excitation patterns (STEP): the internal representation can be treated as a vector of ‘intelligent looks’ or samples of auditory stimuli, with some ‘looks’ weighted more than others for different tasks. Perceptual tasks can be implemented on this type of internal representation^27^.

Recently, based on the results of speech perception experiments as well as neurophysiological studies, one line of argumentation proposes that there are two main temporal windows working concurrently in the auditory system^28^,^29^,^30^,^31^,^32^,^33^: one temporal window, of ~30 ms or less in which acoustic structure is parsed and analyzed with relatively high temporal resolution; and another temporal window of ~200 ms in which acoustic information is extracted at a more global scale. Although such a ‘two-timescale integration model’ has a specific physiological constraint on the size of temporal windows, it is supported by a growing body of neural evidence^30^,^34^,^35^,^36^,^37^,^38^. Its core idea builds on the multiple-looks model and STEP, which is that temporal integration involves a process of sampling or integrating information discretely on a small timescale (e.g, tens of milliseconds) and on a large timescale (e.g. hundreds of milliseconds), instead of simply integrating information continuously, as suggested by the leaky-integration model.

Although the two-temporal-windows integration model (2TWI) shares some basic features with the multiple-looks and STEP models, there are significant differences, as well. For example, 2TWI model suggests that the auditory system may process information differently on different scales^32^. The processing may be modulated by acoustic properties on different timescales or by task requirements so that extraction and representation of acoustic information is more efficient. This leads to predictions different from the ones derived from the multiple-looks and STEP models. For example, in a task comparing local details of sounds, the multiple-looks model and STEP would predict that, as stimulus length increases and hence more local details are available, the auditory system would form more distinct representations. Therefore, the performance, e.g. of differentiating two sounds, should increase with stimulus length. In contrast, the 2TWI model suggests that increased stimulus length may not necessarily increase performance, because representation and processing of local details might function best only at a time range of tens of milliseconds^29^,^32^. On the other hand, the 2TWI model further suggests that representation for global scales is best only for a time range in the hundreds of milliseconds, while the multiple-looks and STEP models would predict the perception of global information increases with increased number of ‘samples’ or ‘looks’ of information.

Here, to explore mechanisms of temporal integration and test certain predictions of the different integration models, we investigated in a series of four psychophysical experiments the perceptual performance at shorter and longer scales by manipulating the timescale of acoustic information while biasing the auditory processes implementing *local* or *global* task demands. Our approach is conceptually analogous to previous local-global studies^39^,^40^. In Experiment 1A, a same-different task is employed to test how various local and global scales affect participants’ performance on abstracting fine-grained, ‘local’ spectrotemporal information as well as more ‘global’ acoustic patterns. In Experiment 1B, by holding fixed the local scale, we further test how stimulus length modulates participants’ ability to extract fine-detailed and global information. Experiment 2 assesses whether the frequency difference at the onset or offset between two stimuli can affect the findings. In Experiment 3, we first measure how much time the auditory system needs to effectively process particular acoustic details (Experiment 3A) and then test whether the integration process functions best over a limited time (Experiment 3B). In Experiment 4, by varying intervals between two stimuli, we ask whether memory factors may affect the results, as the results of the same-different task may be modulated by memory requirements. Table 1 provides an overview of the logic for each experiment and the tasked used in each experiment.

## General Method

### Participants

We conducted all experiments in accordance with procedures approved by the NYU committee on Activities Involving Human Subjects. All participants were from the New York University community and surrounding areas and provided written informed consent before doing experiments. All participants were compensated financially for the time they spent doing experimental tasks.

#### Experiment 1

Twenty-two participants, who were all right-handed and had no known hearing deficit, took part in the experiment. Ten participated Experiment 1A (age range 22 to 33 years; 6 females) and ten participated Experiment 1B (age range 20 to 32 years; 7 females). The data from two subjects were excluded from Experiment 1B because of their failure to follow the instructions.

#### Experiment 2

Twelve listeners participated in this experiment. We excluded the data from two participants because of their failure to comply with the experimental instructions. The remaining 10 participants (age range 18 to 31 years; 6 female) were right-handed and had no reported hearing loss.

#### Experiment 3

Ten and eleven participants, who were right-handed and had no known hearing deficit took part in Experiment 3A (age range 20 to 32 years; 6 female) and Experiment 3B (age range 19 to 32 years; 5 female), respectively. One participant from Experiment 3B did not complete the experiment and therefore was removed from further analysis.

#### Experiment 4

Ten listeners (age range 18 to 31 years; 7 female) participated in the experiment. Nine participants were right-handed and one participant was left-handed according to participant self-report. The participants had no reported hearing loss.

### Stimuli and apparatus

**Figure 1:**
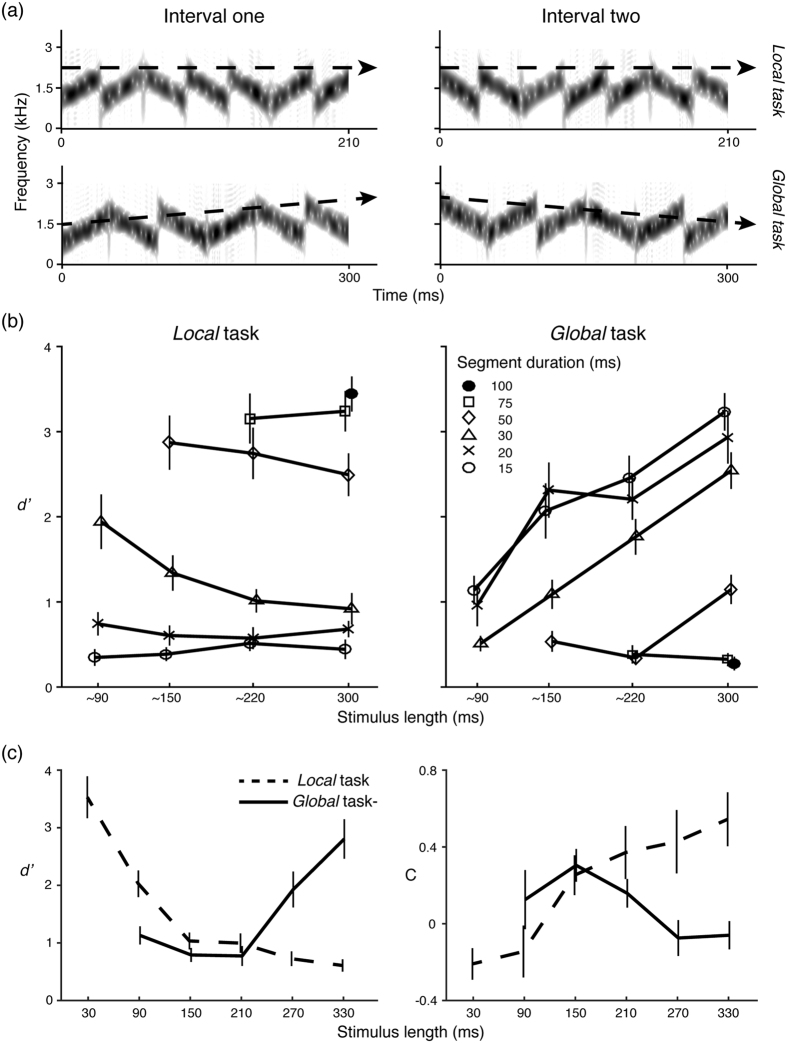
Spectrograms of stimuli and results of Experiments 1A and 1B. (**a**) An example of spectrograms for the ‘different’ pair of stimuli is plotted separately for the *local task* and the *global task*. The dashed arrow schematizes the overall frequency change across stimulus duration. (**b**) Results of Experiment 1A are plotted as a function of stimulus length (x-axis) and d prime (y-axis). The marker shapes represent different segment durations. Longer stimuli do not increase participants’ performance in the *local task* (left), but facilitate recognition of the global sound pattern across different segment durations in *global task (right*). Larger segment duration increases subjects’ performance in the *local task* (left) but decrease the performance in the *global task (right)*. (**c**) The results of Experiment 1B using only segment duration of 30 ms demonstrate the opposite effect of the stimulus length on the *local* and *global* tasks. (**d**) Criteria (**C**) in the *local* and *global* tasks as a function of stimulus length. The criterion in the *local* task increases with stimulus length, which indicates that subjects become worse at analyzing local details and tend to consider two sounds in the ‘different’ pair as the same.

We generated auditory stimuli and presented them to participants using MATLAB (The MathWorks, Natick, MA) and the Psychophysics Toolbox extensions^41^,^42^,^43^. The stimuli were inspired by similar auditory signals used in an fMRI study by Boemio *et al*.^35^ and a magnetoencephalography (MEG) study by Luo & Poeppel (2012). All stimuli were created by concatenating narrow-band frequency-modulated segments, each consisting of a sum of 200 frequency-modulated sinusoids. The amplitude and onset phase of each sinusoid were randomized, and the frequency range was centered at a frequency and varied with the bandwidth of 100 Hz. Each sinusoid was generated individually and all sinusoids were added together to create a narrow-band frequency modulated segment. Therefore, all generated segments spanned the range from 1000 to 2000 Hz with a mean frequency of 1500 Hz and had a bandwidth of 100 Hz, thereby remaining within the critical band at the corresponding frequency. The segments swept linearly upward or downward, randomly over the same frequency range. Each segment in each stimulus was individually generated. Figure 1a,b provide spectrograms of the stimuli.

All stimuli were presented at 16 bit, 44.1 kHz sampling resolution using headphones (Sennheiser HD 380 Professional, Sennheiser Electronic Corporation, Wedemark, Germany). The amplitude was kept at a comfortable level (~60 dB, SPL).

### Analysis Procedures

Responses were transformed into the sensitivity index, d prime, and criterion, denoted as C, as in a simple yes-no paradigm using the Palamedes toolbox^44^.

In Experiments 1, 3 and 4, we used a same-different paradigm. Correct responses to ‘different’ trials were marked as hit and incorrect responses to ‘same’ trials as false alarm. Participants in our experiments may adapt a “differencing” strategy as in the same-different task, since there is no obvious perceptual token in our stimuli as well as stimuli of different segment durations and stimulus lengths were grouped and presented randomly in each task. However, without assuming complex behavior models, treating responses in the same-different paradigm as from a yes-no paradigm can appropriately capture the sensitivity of participants and the behavioral effects caused by experimental modulations, which is the main focus of our study. When a participant responded correctly to all ‘same’ or ‘different’ trials within a block, a half artificial incorrect same or different trial was added to the block^45^. Therefore, the maximum d-prime value for all correct responses is 4.482 (instead of infinity) and the minimum d-prime value is 0.

In Experiment 2, we used a two-interval two-alternative-forced choice paradigm and d prime values were computed accordingly.

## Experiment 1

Both the multi-look and the STEP models predict that longer stimuli provide more local details than similar shorter ones, and hence that perceptual performance increases with stimulus length. In contrast, the 2TWI model hypothesizes that the implementation of different time scales can be modulated by the task demands. Increasing stimulus length does therefore not necessarily enhance perceptual performance on local processing tasks. In Experiment 1A, we manipulate the implementation of local and global scales in different tasks to investigate how local-scale and global-scale processes vary with the timescale of local acoustic details and stimulus length. In Experiment 1B, we further examine how access to fine-temporal-detail information changes with increases in overall stimulus length.

### Method

#### Stimuli and apparatus

In Experiment 1A, two factors, *stimulus length* and *segment duration*, were manipulated. We varied both segment duration and stimulus length and chose specific combinations of number of segments and segment duration to fix overall stimulus length at around 90, 150, 220 and 300 ms. We confined the maximum stimulus length to be 300 ms because, as we observed in pilot data, participants’ performance achieved a ceiling effect or floor effect when stimulus length exceeded 300 ms. The minimum number of segments within a stimulus was set at 3, and the duration of segments was varied at 15, 20, 30, 50, 75 and 100 ms. Therefore, for stimuli of ~90 ms total duration, three segment durations were included, 15, 20 and 30 ms; for stimuli of around 150 ms total duration, 15, 20, 30 and 50 ms segments were included; for stimuli of around 220 ms, 15, 20, 30, 50, and 75 ms; and for stimuli of 300 ms, 15, 20, 30, 50, 75 and 100 ms.

Two tasks, a *local task* and a *global task*, were run in Experiment 1A (Fig. 1a). On each trial, a pair of stimuli was presented. For both tasks, we generated ‘same’ and ‘different’ pairs. In the *local task*, the sweep directions of each corresponding segment were the same between two stimuli in the ‘same’ pair, whereas the sweep directions of each corresponding segment in the ‘different’ pair were opposite. In the *global task*, the sweep directions of corresponding segments between two stimuli were always different because we wanted to prevent participants from making judgments based on acoustic details. In the ‘same’ pair, both stimuli had a mean frequency either increasing from 1300 Hz to 1700 Hz over the stimulus duration or decreasing from 1700 Hz to 1300 Hz; in the ‘different’ pair, one stimulus had a mean frequency increasing from 1300 Hz to 1700 Hz over the stimulus length, and the other one had a mean frequency decreasing from 1700 Hz to 1300 Hz.

In Experiment 1B, segment duration was fixed at 30 ms and the factor ‘stimulus length’ (equivalent to the number of segments) was manipulated in each condition, as Experiment 1A showed that overall stimulus length markedly affected performance of the *local task* at this segment duration. We used the same manipulations as in Experiment 1A: in the *local task*, we generated ‘same’ and ‘different’ pairs of stimuli with varying stimulus length: 30, 90, 150, 210, 270, and 330 ms. In the *global task*, we only selected stimulus length: 90, 150, 210, 270, and 330 ms, because we could not manipulate global pattern when there was only one segment. The sweep directions of corresponding segments between two stimuli were always the same in the *global task* (different from Experiment 1A). In the ‘same’ pair, both stimuli had a mean frequency increasing with the number of segments from 1300 Hz to 1700 Hz, or had a mean frequency decreasing with the number of segments from 1700 Hz to 1300 Hz; in the ‘different’ pair, one stimulus had a mean frequency increasing with the number of segments from 1300 Hz to 1700 Hz, and the other one had a mean frequency decreasing from 1700 Hz to 1300 Hz. Because we wanted to examine how acoustic information is integrated over time to build up a global pattern, we made the mean frequency increase with the stimulus length instead of fixing the range of mean frequency for each stimulus, as we did in Experiment 1A.

#### Procedure and design

The experiment employed a same-different task. On each trial, two stimuli were presented sequentially, with an inter-stimulus interval uniformly distributed from 400 to 600 ms. Participants were prompted to make a response 100 ms after the second interval. The *local task* was to judge whether two stimuli were the same or not. In the *global task*, the participants were asked to judge whether the directions of the mean frequency shift were the same between two stimuli. The *local* and *global* tasks were presented in two separate blocks, and trials within a block were presented in a pseudorandom order. The order of blocks was counterbalanced across participants. In each block, for trials with the same number of segments, there were 40 same trials and 40 different trials for each condition. In Experiment 1A, 1600 trials in total were presented in the *local task* and the same number in the *global task*. In Experiment 1B, 480 trials in total were presented in the *local task* and 400 trials in the *global task*.

Before the formal experiment, three trials for each stimulus length and segment duration in each task were presented (with feedback) to familiarize the participants with the stimuli and tasks.

### Results and Discussion

Figure 1b shows the results of Experiment 1A, mean *d prime value*s as a function of stimulus length (mean ± 1 SEM). As illustrated in the left panel, stimulus length does not modulate sensitivity, as quantified by *d prime value*s, across different segment durations except the segment duration of 30 ms in the *local task* - in which participants needed to compare fine-detailed information between two stimuli. In contrast, *d prime value*s increased with increasing stimulus length in the *global task* in which participants needed to integrate the acoustic information to perceive the global sound pattern. Furthermore, increasing segment duration increases participants’ performance in the *local task* but decreases the performance in the *global task*.

Figure 1c shows the *d prime value*s for Experiment 1B, which demonstrate, again counterintuitively, that increased stimulus length negatively modulated performance in the *local task*. The response criteria, depicted in Fig. 1d, indicate that at increased stimulus length participants tend to smooth out local details and treat two different local stimuli as the same.

In Experiment 1A, we first evaluated how stimulus length affected performance on each task. As the number of stimulus length levels varies across different segment durations (e.g. there are four levels of stimulus length for segment duration of 15 ms while there are only two levels for 75 ms), we conducted a one-way repeated measures ANOVA for each task and each segment duration. We found that stimulus length in the *local task* significantly affected participants’ performance only when segment duration is 30 ms (15 ms: *F*(3,27) = 0.529, *p* = 0.666; 20 ms: *F*(3,27) = 0.419, *p* = 0.741; 30 ms:* F*(3,27) = 14.137, *p* < 0.001; 50 ms: *F*(2,18) = 1.902, *p* = 0.178; 75 ms: *F*(3,27) = 0.210, *p* = 0.658); in the *global task*, the main effects of stimulus length are significant for all levels of segment duration except for 75 ms (15 ms: *F*(3,27) = 35.66, *p* < 0.001; 20 ms: *F*(3,27) = 42.89, *p* < 0.001; 30 ms: *F*(3,27) = 48.28, *p* < 0.001; 50 ms: *F*(2,18) = 15.43, *p* < 0.001; 75 ms: *F*(3,27) = 0.149, *p* = 0.709).

Next we examined the effect of segment duration on different tasks. A two-way repeated measures ANOVA with the factors *task* and *segment duration* was conducted at each level of stimulus length, followed by a one-way repeated measures ANOVA to evaluate the main effect of segment duration within each task at each level of stimulus length. The interaction between task and segment duration is significant for all levels of stimulus length (300 ms: *F*(5,45) = 143.84, *p* < 0.001; ~220 ms: *F*(4,36) = 74.61, *p* < 0.001; ~150 ms: *F*(3,27) = 35.13, *p* < 0.001; ~90 ms: *F*(2,18) = 14.24, *p* < 0.001). The main effect for segment duration is significant when stimulus length is ~220 ms *(F*(4,36) = 2.738, *p* = 0.044) and 150 ms: *(F*(3,27) = 4.285, *p* = 0.013) and ~90 ms: *(F*(2,18) = 25.13, *p* < 0.001). The main effect for task is not significant across all stimulus lengths (300 ms: *F*(1,9) = 0.66, *p* = 0.437; ~220 ms: *F*(1,9) = 1.883, *p* = 0.203; ~150 ms: *F*(1,9) = 1.103, *p* = 0.321; ~90 ms: *F*(1,9) = 0.173, *p* = 0.687). In post-hoc comparisons, the main effect for segment duration is significant in both tasks at all levels of stimulus length (*local task*, 300 ms: *F*(5,45) = 93.33, *p* < 0.001; ~220 ms: *F*(4,36) = 54.10, *p* < 0.001; ~150 ms: *F*(3,27) = 33.02, *p* < 0.001; ~90 ms: *F*(2,18) = 25.85, *p* < 0.001; *global task*, 300 ms: *F*(5,45) = 57.83, *p* < 0.001; ~220 ms: *F*(4,36) = 41.88, *p* < 0.001; ~150 ms: *F*(3,27) = 19.44, *p* < 0.001; ~90 ms: *F*(2,18) = 3.708, *p* = 0.045). Linear trend contrasts showed an upward trend for the *local task* and a downward trend for the *global task*, except when stimulus length was ~90 ms, *F*(1,9) = 2.474, *p* = 0.150.

In Experiment 1B, a two-way repeated measures ANOVA on d prime values with the factors *task* and *number of segments* revealed a significant effect of the number of segments, *F*(4,36) = 14.88, *p* < 0.001, and task, *F*(1,9) = 5.684, *p* = 0.041, and a significant interaction between task and number of segments, *F*(4,36) = 38.54, *p* < 0.001. A one-way repeated measures ANOVA showed a significant effect of the number of segments for both the *local task*, *F*(5,45) = 64.43, *p* < 0.001, and the *global task*, *F*(4,36) = 25.13, *p* < 0.001. Linear trend contrasts showed a downward trend for the *local task*, *F*(1,9) = 99.44, *p* < 0.001, and an upward trend for the *global task*, *F*(1,9) = 25.47, *p* = 0.001. We further investigated how the subjects’ response criterion varied with stimulus length. A two-way repeated measures ANOVA on criterion (C) with the factors of task and number of segments revealed a significant interaction between task and number of segments, *F*(4,36) = 18.81, *p* < 0.001. A one-way repeated measures ANOVA showed a significant effect of the number of segments for the *local task*, *F*(5,45) = 7.47, *p* < 0.001, but not for the *global task*, *F*(4,36) = 2.27, *p* = 0.081. Linear trend contrasts showed a downward trend for the *local task*, *F*(1,9) = 11.18, *p* = 0.009.

The performance on the *local task* shows that differentiating sounds based on detailed, local spectrotemporal information does not increase with increasing stimulus length. According to the multiple-looks and STEP models, longer stimuli provide a larger amount of acoustic information for the auditory system to draw more samples or looks. The auditory system should form more distinct representations of stimuli and lead to better performance. However, the results in both Experiments 1A and 1B suggest that the auditory system fails to (be able to) utilize the extra information provided in the longer stimuli, since performance did not increase. The performance of the *local task* remained at the lowest level across different stimulus lengths when segment duration was short (15 or 20 ms). One possible explanation for the floor effect is that the auditory system cannot resolve detailed information at such short local scales. A ceiling effect may also explain that stimulus length has no effect when segment durations are 50, 75 or 100 ms. At those scales, the listeners can resolve the detailed spectrotemporal information and perceive the sweep directions of the segments. Therefore, the effect of increased stimulus length cannot be observed probably because of a ceiling effect. In contrast, when segment duration was 30 ms, stimulus length exerted a clear negative influence on the *local task* performance, as shown by the main effect of stimulus length in Experiment 1A and again in Experiment 1B. Furthermore, in Experiment 1B, the criterion in the local task increased with stimulus length, which indicates that subjects became worse at analyzing local details and tended to consider two sounds in the ‘different’ pair to be the same. Therefore, these results suggest a more complicated temporal integration process in the auditory system, one that might be consistent with the 2TWI model.

In Experiment 1A, the scale of tens of milliseconds seems to apply to perception of fine-grained information in the *local task*. Performance is poor when segment duration is only 15 ms (mean of d prime value below 1) and becomes very good (and then levels off) when segment duration is 50 ms (mean d prime value greater than 3). To generate a good estimation of local acoustic details, the auditory system may need to integrate information over tens of milliseconds. The range around 30–50 ms seems to be a lower bound for a timescale that facilitates access to such detailed information. These results are consistent with the fact that speech perception requires fine-detailed decoding of phonemic information, which also falls on a scale of tens of milliseconds^1^.

On the other hand, the *global task* shows the reverse pattern, which is predicted by the multiple-looks and STEP models: participants’ performance on the *global task* increases with increasing stimulus duration, arguably because stimuli of longer duration provide more acoustic information, and a global pattern can be better recognized as more information is integrated. In contrast, longer segment duration causes worse performance in the *global task*. When the local scale increases, each stimulus contains fewer segments. Therefore, less information is available for extracting a global pattern.

The results of Experiment 1 are consistent with recent findings by McDermott *et al*.^46^ and Piazza *et al*.^47^, who also showed that increased stimulus length helps the auditory system perceive global statistical patterns but can impede access to local acoustic detail.

## Experiment 2

In Experiments 1A and 1B, participants could in principle use a strategy of monitoring the spectral change of a single segment between two stimuli to respond in the *global task*. That is, subjects could make their responses based on the frequency difference between the onsets or the offsets of stimuli. To address this potential confound, in Experiment 2 we kept the frequency difference between two stimuli at the onset and the offset the same and made all segments different between two stimuli. Only a segment duration of 30 ms is only used in this experiment (as well as in Experiments 3 and 4), because previous behavioral studies^48^ found that frequency modulation sweeps of 30 ms ensure good perception of sweep direction and, therefore, good access to local details. Furthermore, as shown in Experiment 1A, at the segment duration of 30 ms, participants’ performance did not reveal floor or ceiling effects, so a modulatory effect of stimulus length could be well observed.

### Method

#### Stimuli and apparatus

**Figure 2:**
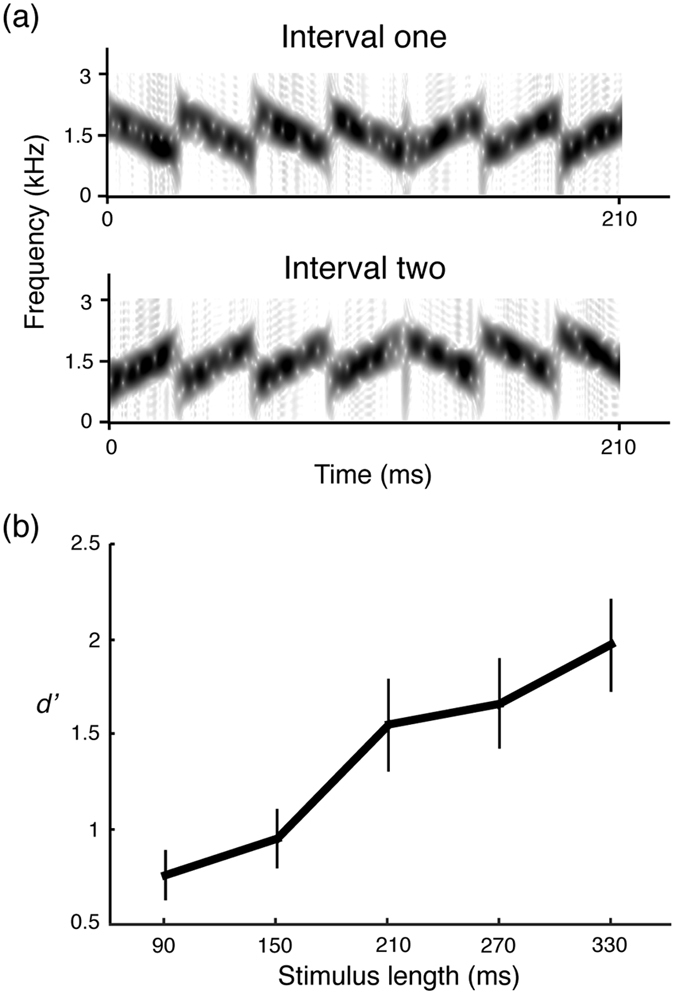
Stimulus spectrograms and results of Experiment 2. (**a**) Examples of spectrograms for the stimulus pair, in which one of the stimuli has flat global mean frequency and the global frequency of the other stimulus shifts upwards. Results of Experiment 2 (**b**) are plotted as a function of stimulus length (x-axis). Although the frequency difference between the two stimuli is kept constant, the ability to recognize global frequency trends increases as a function of stimulus length.

We generated two groups of stimulus pairs, with 3, 5, 7, 9 and 11 segments. The factor *segment number* (similar to *stimulus length* because we fixed the segment duration at 30 ms) was manipulated in two conditions. In *condition 1*, each pair of stimuli included one stimulus with mean frequency shifting from 1300 Hz to 1700 Hz, regardless of the number of segments, and the other stimulus with mean frequency constant at 1500 Hz (Fig. 2a). In *condition 2*, each pair of stimuli included one stimulus with mean frequency shifting from 1700 Hz to 1300 Hz and the other stimulus with mean frequency kept at 1500 Hz (Fig. 2a). The stimuli within a pair always had segments of opposite sweep directions. The frequency difference between two stimuli at the onset was the same as the frequency difference at the offset, so the participants could not rely on spectral cues to judge the trend of the frequency shift. Furthermore, because each segment swept in different directions, participants could not monitor the spectral change of a single segment between two stimuli to generate a response.

#### Procedure and design

We used a two-interval two-alternative-forced choice paradigm and block design for each condition. Participants reported which interval shifted upward in *condition 1*, and which interval shifted downward in *condition 2*. For each number of segments, 30 trials were presented (total of 720 trials).

### Results and Discussion

Figure 2b summarizes the results: as the number of segments (i.e. stimulus length) increased, *d prime value*s increased. A two-way repeated measures ANOVA revealed that the main effect was significant for number of segments *F*(4,36) = 23.07, *p* < 0.001, but not for shifting direction *F*(1,36) = 2.35, *p* = 0.160. There was no interaction *F*(4,36) = 1.83, *p* = 0.145. The results show that, when the frequency differences of onset and offset between two stimuli were kept the same for all stimulus length, it became very difficult for participants to identify which interval had a frequency shift when sound duration was short (<150 ms). Therefore, this experiment rules out the possibility that the results of Experiment 1 were caused by the frequency difference between the stimuli.

## Experiment 3

In the *local task*, longer stimulus length did not increase participants’ performance. One explanation is that more segments require more processing time to represent local details. Within a short period of time, the acoustic details of only several segments can be fully accessed. When the amount of information extends beyond the capacity of processing within a certain time window, the auditory system ‘summarizes’ the local details and forms a representation of a global pattern. Another explanation is that increased stimulus length increases the amount of information, namely the number of segments, carried in the working memory. The increased working memory load may potentially degraded participants’ performance in the *local* task. In this experiment, we keep the number of segments constant while inserting intervals between segments, to assess whether local details can be recovered by providing more processing time. If the working memory load plays a key role here, inserting intervals while keeping the memory load consistent will not change the participants’ performance.

We hypothesize that acoustic information can only be integrated within a limited temporal range. By keeping constant the information provided by stimuli (the number of segments) while increasing stimulus length by inserting gaps between segments, we can test whether the auditory system can store all segments and integrate acoustic details over a longer time range. If the acoustic information can be integrated without a time constraint, the stretched stimuli will not affect the performance in the *global task*. Otherwise, the performance will decrease as the size of intervals increases.

In Experiment 3A, we examine the amount of additional time the auditory system needs to recover fine-detailed information. In Experiment 3B, we ask whether temporal integration functions best on a time scale of hundreds of milliseconds. We fix both the number of segments and segment duration while manipulating overall stimulus length by inserting gaps between segments.

### Method

#### Stimuli and apparatus

**Figure 3:**
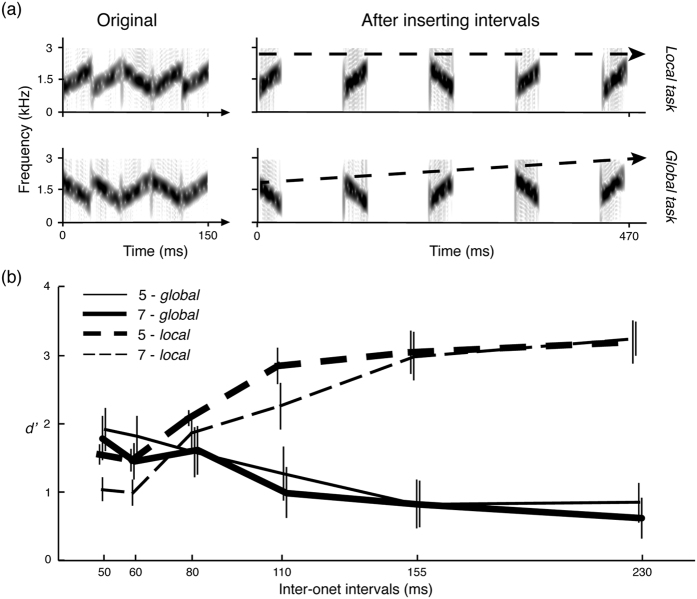
Spectrogram of stimuli and results of Experiment 3A and 3B. (**a**) The left panel shows the spectrograms of the original stimuli for the *local* and *global* tasks, and the right panel shows the spectrograms of the stimuli after the intervals are inserted. (**b**) The results as a function of inter-onset intervals (x-axis) and d prime value (y-axis). The dashed line represents results of Experiment 3A, the *local* task and the solid line represents results of Experiment 3B, the *global task*. As the inter-onset interval increases, it facilitates access to the fine-detailed information, while the ability to recognize the global sound pattern decreases because acoustic segments fall out of the putative temporal integration window.

Two additional factors, the number of segments and inter-onset interval (IOI), were included in this experiment. The IOI is defined here as the duration between the onset of one segment and the onset of the next segment (see Fig. 3a).

In Experiment 3A, segment duration was fixed at 30 ms and stimuli with 3 and 5 segments were used. Six levels were used for the factor IOI: 50, 60, 80, 110, 155, and 230 ms. Half of trials were ‘same’ and half were ‘different’ pairings. A large IOI may make the first and the last segments more salient than middle segments, because no segment precedes the first segment and no segment follows the last segment. Participants may just pay attention to the first or the last segment to do the tasks instead of resolving acoustic details of the whole stimulus. To avoid this potential confound, the sweep directions of the segments in the beginning and the end of the stimuli were the same between two stimuli in both ‘same’ and ‘different’ pairs; the segments in the middle of the stimuli were the same in the ‘same’ pair, and different in the ‘different’ pair.

In Experiment 3B, stimuli with 5 and 7 segments were used. The same six levels were used for the factor IOI. The stimuli within both ‘same’ and ‘different’ pairs always had segments of opposite sweep directions. For ‘same’ pairs, regardless of the number of segments, the stimuli either had a mean frequency increasing with the number of segments from 1300 Hz to 1700 Hz or a mean frequency decreasing from 1700 Hz to 1300 Hz. For ‘different’ pairs, the stimuli had opposite directions of frequency shifting trend.

#### Procedure and design

Stimuli with different number of segments were presented in separate blocks, and in each block the order of IOIs was randomized. The order of the blocks was counterbalanced across participants. For each IOI, the stimuli were the same for 30 trials, and different for 30 trials. In total, 720 trials were presented for Experiment 3A and Experiment 3B, respectively. Around ten trials for each number of segments at each IOI were presented with feedback for training purposes before the experiment.

### Results and Discussion

Figure 3b shows that *d prime value*s increased as the IOIs increased in the *local task* (Experiment 3A) but decreased in the *global task* (Experiment 3B). A two-way mixed factor ANOVA with *task* (the *local task* in Experiment 3A and the *global task* in Experiment 3B) as a between-subject factor and *IOI* as a within-subject factor revealed significant main effects of task, *F*(1,18) = 6.37, *p* = 0.021, and IOI, *F*(5,90) = 5.739, *p* < 0.001, and a significant interaction, *F*(5,90) = 55.13, *p* < 0.001.

In Experiment 3A, *d prime value*s increased as the IOIs increased. A two-way repeated measures ANOVA shows that the main effects of the number of segments *F*(1,9) = 10.80, *p* = 0.001 and the IOI, *F*(5,45) = 43.89, *p* < 0.001 were significant and that the interaction between the number of segments and the IOIs was also significant, *F*(5,45) = 2.83, *p* = 0.027. In the post-hoc test, we found significant upward linear trend for both 3 segments, *F*(1,9) = 72.22, *p* < 0.001, and 5 segments, *F*(1,9) = 48.30, *p* < 0.001.

In Experiment 3B, *d prime value*s decreased as the IOIs increased. A two-way repeated measures ANOVA showed that the main effect was significant for IOI, *F*(5,45) = 14.99, *p* < 0.001, but not for the number of segments, *F*(1,9) = 2.34, *p* = 0.161. No significant interaction effect was found, *F*(5,45) = 0.88, *p* = 0.504. Therefore, we combined the data from trials with different number of segments into one dataset, and the linear trend contrasts showed a downward trend *F*(1,9) = 32.87, *p* < 0.001.

The results from Experiment 3A suggest that the auditory system requires time to process and represent fine-grained information. The intervals between segments and, of course, the duration of segments are both important for extracting fine-grained information from the stimuli. Similar results have been found in a speech perception study^49^ in which inserting intervals into compressed speech increased intelligibility. Contrary to the results of Experiment 1B, it seems that participants in this experiment are better at the *local task* using 5-segment stimuli than 3-segment stimuli. We suspect that the discrepancy is caused by the fact that we fixed the first and the last segments in Experiment 3A, so differences between two stimuli within the ‘different’ pair are much smaller in 3-segment stimuli (only one segment is different) than in 5-segment stimuli (three segments are different).

The results from Experiment 3A also demonstrate that the working memory load cannot explain the effect of stimulus length in the *local* task of Experiment 1. By inserting intervals into the stimuli, we kept the working memory load constant and stretched the stimuli to make the task more memory demanding, but the participants’ performance became better rather than worse. Therefore, the effect of stimulus length is related more to properties of auditory processing than to effects of the working memory load.

Importantly, in Experiment 3B, the ability to recognize the global sound pattern decreases as IOI increases. Grouping acoustic details and abstracting a global pattern seem only to work well within a particular, restricted time range. Only if the necessary information falls within a short time range (e.g. hundreds of milliseconds), can the auditory system integrate acoustic information and abstract the global trend. Otherwise, the stimuli start to sound like a sequence of individual acoustic segments instead of a coherent, integrated sound pattern, so local details become salient and could potentially affect the integration process.

## Experiment 4

In the *local task* of Experiment 1, the same-different judgment of two stimuli based on fine-grained information may be influenced by memory constraints. That is, when we varied stimulus length, this lengthened the amount of time participants needed to remember information about the first stimulus in the stimulus pair. If memory degraded during that time, this may have been one factor contributing to the effect of stimulus length. To examine this hypothesis, the interval between two stimuli within a pair was varied in Experiment 4. If memory plays an important role in the *local task* of Experiments 1A and 1B, performance should decrease as the interval between two stimuli increases.

### Method

#### Stimuli and apparatus

The factors, segment number (i.e. overall stimulus length) and inter-stimulus interval (ISI), were manipulated. We generated stimulus pairs with 1, 3, 5, and 11 segments. ISIs were varied across four levels: 500, 700, 1000 and 1500 ms.

#### Procedure and design

The procedure was the same as in the *local task* of Experiment 1. Pairs of stimuli with different segments and ISIs were pseudo-randomly presented. All stimuli were presented in one block. For each number of segments and each ISI, 30 trials were presented, for a total of 960 trials. Around ten trials for each number of segments at each ISI were presented with feedback for training purposes before the formal experiment.

### Results and discussion

**Figure 4:**
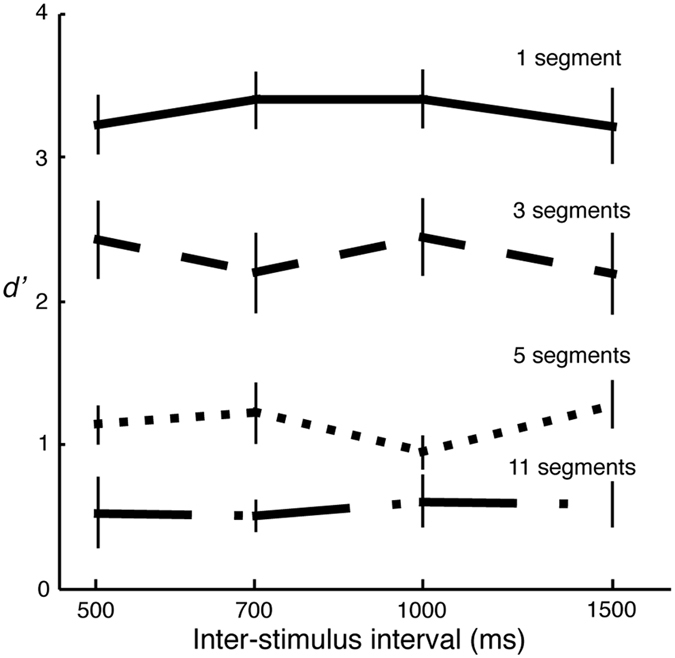
Results of Experiment 4. The d prime values do not vary with inter-stimulus intervals, which suggests that memory factors do not affect the results in Experiment 1. Different line types represent stimuli with different number of segments.

Figure 4 illustrates that *d prime value*s did not change across different ISIs for a given stimulus length. A two-way repeated measures ANOVA shows that the main effect was significant for number of segments *F*(3,27) = 94.51, *p* < 0.001, but not for ISIs *F*(3,27) = 0.06, *p* = 0.982. There was not enough evidence to show a significant interaction effect *F*(9,81) = 1.76, *p* = 0.090. These results are consistent with previous work in which the interval between stimuli is varied systematically to examine the effect of auditory memory; no significant effect of interval between stimuli is found^50^. Although memory requirements may come into play in our experiment due to the characteristics of the same-different procedure, the results of this control experiment demonstrate that memory cannot explain the main results obtained in the previous experiments.

### General Discussion

We report the findings from four behavioral studies designed to investigate how auditory information on different timescales is integrated. Experiment 1 showed that the auditory system can extract fine-temporal-detail information as well as integrate acoustic chunks to form a global percept. However, longer stimuli only improved integration in a *global task*, but did not increase performance on extracting fine-grained information in a *local task*. Experiment 2 suggested that potential frequency difference cues between stimuli did not affect the results of temporal integration obtained in Experiment 1. Experiment 3A showed that decoding and representing the detailed acoustic information required processing time. Experiment 3B suggested that temporal integration functions best within a limited time range. Experiment 4, finally, controlled for the possibility that memory factors affected the conclusions of Experiment 1.

How the auditory system extracts fine-grained information and integrates acoustic information over time remains a key issue for auditory processing. Recent studies by McDermott *et al*.^46^,^51^ and Piazza *et al*.^47^ argue that the auditory system uses a form of statistical summation to integrate acoustic information over time to generate auditory percepts, but the temporal range of putative statistical summation processes in these studies was not clearly articulated. Durations of the stimuli used in McDermott *et al*.^46^ range from 40 ms to more than 2 seconds, and results similar to our findings were found: when discrimination depends on the details of the stimuli, performance decreases with sound duration; when discrimination depends on the long-term statistics of the stimuli, performance increases with sound duration. In Piazza *et al*.^47^, the sound duration used is more than two seconds, which is a different time range than that used in the present study. In both studies, short-term memory may be involved in the tasks, so the summation process cannot be defined with certainty as a characteristic of the auditory system independent from additional memory factors.

In Experiment 1 of the present study, the stimulus length was varied from 30 ms to 330 ms. The data from Experiment 1A demonstrated that access to the fine-grained information of the stimuli improves as segment duration increased over tens of milliseconds - but deteriorates with increased stimulus length. The results of Experiment 1B showed that the representation of the global pattern increased to about 200 ms. These results are consistent with the psychophysical studies that reported two time constants in the auditory system^8^. Moreover, these results suggest that auditory processing is different on these two timescales. On the timescale of hundreds of milliseconds, acoustic information can be integrated and well represented while the representation of fine-grained information is compromised; on the timescale of tens of milliseconds, the acoustic information is processed but arguably not ‘fully’ represented to subserve subsequent perceptual analysis.

The results cannot be explained in a straightforward manner by models assuming a standard process such as a leaky integrator, because the stimuli in the present study have a multiplexed temporal structure, and the task does not require integration of either the envelope or the sound energy. Although the multiple-looks model implies that the auditory system samples acoustic information on a short timescale, the details of the sampling process are not specified. Furthermore, as stimulus length increases, there should be more opportunities for sampling acoustic information. However, we find that the performance of discrimination between two stimuli with different fine-grained information decreases, which is inconsistent with the prediction of a multiple-looks model and a STEP model. The findings of the present experiments suggest a more complicated temporal integration process in the auditory system: the acoustic segments are analyzed within a short temporal window for extracting local details and concurrently acoustic information is integrated within a larger temporal window to form a global percept.

In Experiment 3, access to fine-detail acoustic information is recovered if sound segments are followed by a silent interval of hundred milliseconds. This suggests that the representation of fine acoustic information can only be well-formed within a temporal window of this range. The results enrich explanations of a recent study of speech perception^49^, in which compressed speech becomes more intelligible after inserting intervals between segments, and also echo earlier findings (for a review, see Massaro, 1972). It is worth bearing in mind that, in a range of neurophysiological studies, ~150–300 ms are around the length of one cycle of neural oscillations in the theta band (~3–7 Hz) that are found in speech perception and other auditory tasks^30^,^31^,^34^,^36^,^37^,^52^,^53^. Experiment 3 provides behavioral evidence that the auditory system chunks incoming information into units of the appropriate temporal granularity^29^.

Inspired by the multiple-looks and STEP models, we suggest a slightly different temporal integration process to account for the present results. We hypothesize that two stages of temporal integration exist in the auditory system. In the first stage, the acoustic information is sampled using two temporal windows, a local one in the tens-of-milliseconds range and a global one in the hundreds-of-milliseconds range. Detailed information is sampled by the local temporal window so that fast fluctuations and fine-grained information can be analyzed in a high-time-resolution manner; in contrast, acoustic patterns or statistics, such as pitch and loudness, are better abstracted using the global temporal window so that robust estimation of these acoustic attributes can be achieved. In a second stage, the sampled information is channeled to a representational stage with limited information capacity and probably with a time constant of hundreds of milliseconds. The information at this representational stage supports making responses and memory storage. Because it is unlikely that all detailed information can be represented or stored, the representation of sounds may not simply be an entire set of acoustic samples. The information is ‘smoothed’ or sparsely coded. Attention driven by task demands at this stage can bias representation so that only selected information is represented.

Such a proposed integration model can explain four main findings in the present study:

1. Longer stimulus length does not increase performance in the *local task* as demonstrated by Experiment 1. Although the local details can be well sampled as suggested by the multiple-looks model and STEP using a short temporal window, the representation of the local details is not a vector of samples, as STEP suggests. When the segment size is fixed, a longer stimulus means more samples of segment directions are presented. However, the limited information capacity of the representation stage forces the auditory system to smooth or summarize all information. The details are lost at the representation stage, if too much information is squeezed into the representation. In contrast, when there are only a few segments in the stimuli, the amount of detailed information is low and the information can be well represented, since no ‘smoothing’ or ‘summarizing’ process is implemented. Therefore, increasing stimulus length does not improve performance, and the performance in the *local task* is best at short stimulus length.
2. Global perception is best in the range of hundreds of milliseconds. The auditory system employs a long temporal window to abstract information on a global scale at the first stage and, depending on task requirements, represents global patterns in this representation stage. This contrasts with models suggesting that a global process is increasingly accumulating information derived from a short integration scale. This can explain a sharp change of performance at around 150 ms of stimulus length in the *global task* of Experiment 1B. As stimulus length increases to a point, a global pattern emerges and is sampled by the long temporal window. When the segment duration is varied, the small segment duration (<50 ms) does not affect the global percept because of the direct sampling on a global scale. When segment duration increases above 75 ms, although each segment still carries information of global trend, local directions of segments are sampled by long temporal window so the recognition of global pattern decreases.
3. An interval is needed for access to local details shown by Experiment 3. Given a certain amount of time, the representational capacity is limited. After intervals are inserted between segments, information per unit time decreases, so local details can be represented in a more precise manner. Therefore, the performance in the *local task* of Experiment 3A gets better as the length of intervals increases.
4. Inserting intervals decreases performance in a *global task* as in Experiment 3B. As the long temporal window is around hundreds of milliseconds, inserting intervals stretches the stimuli and makes few segments fall within one long temporal window, so a global pattern of the stretched stimuli cannot be sampled.

## Conclusion

Given the results of the present study, the closest heuristic model that accounts for the data is a two-timescale model: one temporal window works on short timescale (<50 ms) to resolve and integrate fine-grained information; concurrently another window works on a long timescale (~200 ms) to extract the global pattern of sound segments. Such a multi-time resolution hypothesis, proposed by Poeppel (2001, 2003) for speech is probably a general mechanism for audition, not just a specific model for speech perception as was originally argued. Our results thus build on the previous models of temporal integration and shed light on the multiple timescales of auditory processing.

## Additional Information

**How to cite this article**: Teng, X. *et al*. Testing multi-scale processing in the auditory system. *Sci. Rep.* **6**, 34390; doi: 10.1038/srep34390 (2016).

## Figures and Tables

**Table 1:** An overview of the current study.

	Motivation	Task
Experiment 1A	We varied both the segment duration and the stimulus length to investigate how acoustic information on the local and global scales affects auditory processing on both the *local* and *global* tasks. We predict that the auditory system requires tens of milliseconds to resolve information on the local scale while hundreds of milliseconds are required for the global scale. This may reveal a two-scale processing mechanism in the auditory system.	Same-different
Experiment 1B	We fixed the segment duration at 30 ms while varying the stimulus length to further demonstrate the modulation effect of the stimulus length observed in Experiment 1A on both the *local* and *global* tasks.	Same-different
Experiment 2	We controlled the global frequency shift range of the stimuli and used a different paradigm. By doing this, we can test whether the frequency shift range changes our interpretation of our results in Experiment 1.	Two-alternative-forced choice
Experiment 3A	The effect of stimulus length in the *local* task of Experiment 1 could be caused by the capacity limit of information processing or the increased working memory load. We test these two possibilities by inserting intervals between segments to decrease the amount of information per unit of time while keeping the memory load constant.	Same-different
Experiment 3B	By inserting gaps between segments, we test whether the auditory system can store all segments and integrate acoustic details over a longer time range.	Same-different
Experiment 4	When we varied the stimulus length in the *local* task, this lengthened the amount of time participants needed to remember information about the first stimulus in the stimulus pair. If memory degraded during that time, this may have been one factor contributing to the effect of stimulus length in the *local* task. To examine this hypothesis, the interval between two stimuli within a pair was varied in Experiment 4.	Same-different
